# Correction: Effective maternal, newborn and child health programming among Rohingya refugees in Cox’s Bazar, Bangladesh: Implementation challenges and potential solutions

**DOI:** 10.1371/journal.pone.0234227

**Published:** 2020-05-29

**Authors:** Malabika Sarker, Avijit Saha, Mowtushi Matin, Saima Mehjabeen, Malika Asia Tamim, Alyssa B. Sharkey, Minjoon Kim, Elévanie U. Nyankesha, Yulia Widiati, A. S. M. Shahabuddin

The affiliation for the tenth author is incorrect. A. S. M. Shahabuddin is not affiliated with #4 but with #3: Implementation Research and Delivery Science Unit, Health Section, UNICEF Headquarters, New York, NY, United States of America.
